# Beyond morbidity and mortality: the burden of infectious diseases on healthcare services

**DOI:** 10.1017/S0950268819001298

**Published:** 2019-08-07

**Authors:** Edoardo Colzani

**Affiliations:** European Centre for Disease Prevention and Control (ECDC), Solna, Sweden

The impact of infectious diseases on population health has been traditionally measured by the number of cases and deaths attributable to a specific infection. This information, if accurate and representative of the target population, provides an estimate of the overall health burden of a disease, and is most suited to assessing diseases that lead to either rapid recovery or death. For other infectious diseases though, the infection-related long-term consequences such as disabilities and the subsequent loss of quality of life, considerably contribute to the overall burden of disease as well. Collection of morbidity data on disease sequelae and impact on quality of life is more complex, and less directly measurable, than collection of data on disease occurrence and mortality, and is also subject to many assumptions and judgements [1]. Different indicators and sources of data can be used, as Morbey *et al*. have shown investigating the burden of respiratory illness in England by using a national telehealth system. When measuring the health burden of a disease however, it is often required to deal with large uncertainties related to the estimation of the total number of cases, attribution of mortality and attribution of disabilities associated with the disease and its sequelae, among others [2]. Nevertheless, as concluded by Walker *et al*., the availability of estimates of disease burden across all healthcare settings can inform prevention policies by, for instance, estimating the averted cost caused by the disease if the burden is reduced, or the cost-effectiveness of an intervention like varicella vaccination.

Increasingly, the impact and burden of disease beyond individual health is being considered. Social and economic consequences are well-recognised externalities related to the spread of an infectious disease in a population. For example, Buck *et al*. estimated that the excess cost of pertussis patients with previous chronic obstructive pulmonary disease and asthma in the USA was $6154 and $1639 over a 6 month period before and after pertussis diagnosis, while Park *et al*. calculated that the total socioeconomic cost for the treatment of anogenital warts increased 2.6 times between 2007 and 2015 in South Korea.

Another important dimension frequently overlooked in the past is the burden of a disease on healthcare services. Increased incidence and outbreaks can have resource and capacity implications for disease management, clinical and public health practice, and introduce additional operational burden on healthcare systems whose impact, consequences and costs need to be appropriately understood and analysed [3]. The expression ‘burden of disease’ could thus be intended in a broader sense as a measurement of the impact of a disease on population health, healthcare services, society and economy as a whole [4]. While there are continuous commendable calls for data availability and data quality to guide public health action, it should be equally emphasised that these efforts may pose additional operational burden on healthcare workers [5]. Mook *et al*. showed that also the introduction of technologies, such as whole genome sequencing for all presumptive Salmonella isolates, may cause additional operational burden on health protection services despite providing added value. Measuring the operational burden of infectious diseases, as performed by Niang *et al*., concerning sentinel surveillance clinics visits for influenza-like illness in Senegal and by Morbey *et al*., in relation to respiratory tract infection consultations in England, may thus help find system solutions for healthcare planning. Although the measurement of the burden of infectious diseases on healthcare services could imply new technical challenges and sources of uncertainty, the monitoring and reporting of the impact of infectious diseases in all its dimensions with a comprehensive and quantitative approach is crucial for reaching a well-informed decision making.

At the policy level there is a growing demand for evidence for planning and prioritising interventions which goes beyond morbidity and mortality and looks more into healthcare system performance. This is in particular true in high-income countries where the individual health burden of infectious diseases has reduced. The burden of infectious diseases on healthcare services is thus a key factor to be increasingly taken into consideration, as changes in clinical or public health practice and in disease trends may increase costs, introduce unforeseen externalities and add strain to the healthcare system affecting its performance.
